# Impact of two *myostatin *(*MSTN*) mutations on weight gain and lamb carcass classification in Norwegian White Sheep (*Ovis aries*)

**DOI:** 10.1186/1297-9686-42-4

**Published:** 2010-01-29

**Authors:** Inger A Boman, Gunnar Klemetsdal, Ola Nafstad, Thor Blichfeldt, Dag I Våge

**Affiliations:** 1 Department of Animal and Aquacultural Sciences, Norwegian University of Life Sciences (UMB), PO Box 5003, N-1432 Ås, Norway; 2 The Norwegian Association of Sheep and Goat Breeders, PO Box 104, N-1431 Ås, Norway; 3 Animalia - Meat and Poultry Research Centre, PO Box 396 Økern, N-0513 Oslo, Norway; 4 Centre for Integrative Genetics (CIGENE), Department of Animal and Aquacultural Sciences, Norwegian University of Life Sciences (UMB), PO Box 5003, N-1432 Ås, Norway

## Abstract

**Background:**

Our aim was to estimate the effect of two *myostatin *(*MSTN*) mutations in Norwegian White Sheep, one of which is close to fixation in the Texel breed.

**Methods:**

The impact of two known *MSTN *mutations was examined in a field experiment with Norwegian White Sheep. The joint effect of the two *MSTN *mutations on live weight gain and weaning weight was studied on 644 lambs. Carcass weight gain from birth to slaughter, carcass weight, carcass conformation and carcass fat classes were calculated in a subset of 508 lambs. All analyses were carried out with a univariate linear animal model.

**Results:**

The most significant impact of both mutations was on conformation and fat classes. The largest difference between the genotype groups was between the wild type for both mutations and the homozygotes for the c.960delG mutation. Compared to the wild types, these mutants obtained a conformation score 5.1 classes higher and a fat score 3.0 classes lower, both on a 15-point scale.

**Conclusions:**

Both mutations reduced fatness and increased muscle mass, although the effect of the frameshift mutation (c.960delG) was more important as compared to the 3'-UTR mutation (c.2360G>A). Lambs homozygous for the c.960delG mutation grew more slowly than those with other *MSTN *genotypes, but had the least fat and the largest muscle mass. Only c.960delG showed dominance effects.

## Background

In Norwegian White Sheep (NWS), two *myostatin *(*MSTN*) mutations affecting conformation and fat classes are segregating: the 3'-UTR mutation creating an illegitimate microRNA site (c.2360G>A) that was identified in Texel sheep [1] and a frameshift mutation explained by a deletion of one base pair in nucleotide position 960 (c.960delG), identified in NWS [2]. While c.2360G>A reduces the level of circulating myostatin to approximately one third, c.960delG generates a completely non-functional protein.

Initially, the aim of the current study was to investigate the effect of the c.960delG mutation on growth and carcass traits in NWS under ordinary commercial management conditions. NWS is a synthetic crossbreed, composed of the Dala, Rygja, Steigar and Texel breeds [3]. However, during the course of this experiment, another *MSTN *mutation (c.2360G>A) was published [1]. Since the Texel breed is one of the NWS founder breeds [3,4], the ongoing study was expanded in order to include this new mutation. Here we present data on how the two mutations affect weight gain and lamb carcass classification.

## Methods

### Genotyping

Genotyping of the two *MSTN *positions, c.960 and c.2360, was carried out as described by Boman et al. [2]. First, the animals were genotyped only at position c.960, and then retyped at position c.2360, after publication of the second mutation.

### Experimental design

The field experiment comprised two experimental years in the Vesterålen area, in the north of Norway.

#### Year 1

The first year, all ewes of ten commercial NWS flocks were genotyped at the c.960 position. In essence, for each ewe homo- or heterozygous for c.960delG, an age-matched control ewe without the mutation from the same flock was also included in the study. All ewes were mated to a ram without the mutation (n = 34). Two flocks were excluded from the study due to the low numbers of ewes carrying the mutation (4 and 6, respectively). The remaining flocks were genetically well tied, since six belonged to the same ram circle, one was a former member of the circle and one had a history of rams purchased from the circle. A total of 200 ewes (100 case/control pairs) were included in the study, and each flock was represented with 18 to 28 ewes. In six flocks, ultrasound scanning to count the number of foetuses had been performed, thus only pregnant ewes were included in the experiment. The first priority was to include all homozygous ewes, thereafter the youngest heterozygous ewes within each flock. The numbers of ewes and rams per genotype are given in Table 1. The selected ewes' lambs born this year were genotyped.

**Table 1:** Number of ewes and rams (local/AI) per genotype and year

Sex	Ewes sampled			Rams		
		
c.960	GG	G(delG)	(delG)(delG)	GG	G(delG)	(delG)(delG)
Year 1	100	96	4	29/5		
Year 2	101	96	3		10/7	1/0

Guanine (G) is found in the mutated position (c.960) in the wild type; in year 2, a local ram serviced ewes that returned

#### Year 2

It was decided to replace two of the flocks from year 1, by another flock. This flock was in an adjacent ram circle, having genetic ties to the original experimental flocks because common AI rams had been used and local elite rams had been exchanged. Basically, the same sampling strategy as in year 1 was followed; 100 ewes with the c.960delG mutation and 100 without were included. In both groups, ewes with a low estimated overall breeding value were sampled, since these are not relevant for producing replacements. Prediction of the breeding value, is described by Eikje et al. [5]. Each flock was represented with 20 to 30 ewes. In addition, we balanced the groups with respect to age and flock as in year 1. All ewes were artificially inseminated with frozen semen from rams heterozygous for the c.960delG mutation (n = 7). For the ewes that returned, a local ram carrying the mutation was used. The numbers of ewes and rams per genotype are given in Table 1. The selected ewes' lambs were also genotyped in year 2.

### Management and slaughter

The experiment did not interfere with normal management; for example, the farmers were allowed to move lambs to a foster mother or providing supplemental feeding. In year 1, the farmers decided if and when to slaughter the lambs, while in year 2 all experimental lambs were intended to be slaughtered.

At approximately four months of age, the lambs were gathered and transferred from the rough grazing pasture to the farm. Subsequently, the weaning weight of the lambs was measured and the farmers selected the lambs to be sent directly for slaughter, and those to be kept on rich pasture, for finishing. Live weight was used as a guide to decide when to slaughter the lambs according to common practise. Some farmers shipped lambs only twice in the season, while others shipped them more frequently, depending on management choices and flock size.

The lambs were all slaughtered in the same commercial abattoir, and carcass classification was carried out according to the EUROP classification system in Norway [6], which is on a 15-point scale, a value of 15 being the meatiest or fattiest class, respectively.

### Statistical analysis

Data on growth and carcass traits were retrieved from the national sheep recording system (SRS). The data were analysed univariately for weight gain per day from birth to weaning, weaning weight, carcass weight gain per day from birth to slaughter, carcass weight, carcass conformation class and fat class (Y_ijklmno_), with the following linear model, using DMUAI in the DMU software package [7]:

where G_i _is the fixed effect of the ith genotype class (1, ..., 6; see Table 2), GD_j _is the fixed effect of the jth genotype class of the dam (1, ..., 5; as in Table 2, except the class homozygous for c.960delG), S_k _is the fixed effect of the kth sex class (male or female), R_l _is the fixed effect of the lth rearing class (1, 2, ≥3 or bottle lamb), AD_m _is the fixed effect of the mth age of dam class (1, 2, 3, 4 or ≥5), fy_n _is the random effect of the nth flock-year class (1, ...., 15), i_o _is the random additive genetic effect of the oth animal and e_ijklmno _is the random residual term. The pedigree file comprised a total of 3292 animals, a pruned subset retrieved from the SRS for the experimental animals, comprising all known ancestors in six generations.

In the statistical model, the effects of sex, rearing class and age of dam were factors that we *a priori *believed to affect the traits since they are taken into account in the national prediction of breeding values for traits recorded in the autumn.

**Table 2:** Number of lambs per genotype group for various traits

c.960	GG			G(delG)		(delG)(delG)
			
c.2360	GG	AG	AA	GG	AG	GG
Weigth gain/d from birth to weaning (g)	78	216	114	105	106	19
Weaning weight (kg)	78	219	114	107	107	19
Carcass weight gain/d from birth to slaugther (g)	59	165	84	92	89	15
Carcass traits	59	167	84	94	89	15

Guanine is found at the mutated position in wild types, both in the c.960 and the c.2360 position, while (delG) and adenine (A) respectively, are found when the mutations are present. Carcass traits are carcass weight, carcass conformation class and carcass fat class.

An equivalent model, analysing the same data with the same software, was used to estimate the allelic effects rather than the genotype class effects:

where the regression coefficients for the additive and dominant allelic effect of c.2360G>A (a_2360_, d_2360_) and c.960delG (a_960_, d_960_) are given as well as their interaction (int), while the x'es are indicator (dummy) variables; x_1 _is the number of c.2360G>A alleles (0, 1, 2), x_2 _is 1 if heterozygous in c.2360 and 0 otherwise, x_3 _is the number of c.960delG alleles (0, 1, 2), x_4 _is 1 if heterozygous in c.960 and 0 otherwise, x_5 _is 1 for compound heterozygotes and 0 otherwise, and the other terms are defined as in the model above.

To test the impact of the two *MSTN*-mutations in the first model, the wild type individuals (GG_GG, for cDNA position 960 and 2360, respectively) were used as reference. We also wanted to test the impact of the genotypes carrying the c.960delG-mutation, against the group GG_AA. Hypothesis testing was done by the following contrasts, using V3.1 of PEST [8], with variance components from the DMUAI run as input:

1. H_0_: MSTN-genotype - GG_GG (wild type) = 0,

where MSTN-genotype is GG_AG, GG_AA, G(delG)_GG, G(delG)_AG or (delG)(delG)_GG against H_1_: MSTN-genotype - GG_GG (wild type) ≠ 0.

2. H_0_: MSTN-genotype - GG_AA = 0,

where MSTN-genotype is G(delG)_GG, G(delG)_AG or (delG)(delG)_GG, against H_1_: MSTN-genotype - GG_AA ≠ 0.

Hypothesis testing for the allelic effects in the second model was done by the following contrasts, using the same software and variance components:

1. H_0_: regression coefficient = 0,

where regression coefficient is the additive, dominance and interaction terms a_2360_, d_2360_, a_960 _d_960 _and int, against H_1_: regression coefficient ≠ 0.

2. H_0_: a_960 _- a_2360 _= 0,

against H_1_: a_960 _- a_2360 _≠ 0.

Note that since the two models are equivalent, some of the tests are identical.

Estimation of variance components for daily carcass weight gain did not converge due to little information in the data. The heritability was therefore set to 15%.

## Results

The number of homozygous c.960delG ewes was low (Table 1), and thus their progeny were omitted from the analysis. In the autumn, 644 lambs (50.9% females) were recorded with weaning weight (Table 2) and 508 were slaughtered. However, due to recruitment, only 41.2% of the slaughtered lambs were females. The mean age of the dams was 3.1 years, ranging from 1 to 7 years. The average number of lambs weaned was 2.3, ranging from 1 to 4. Eleven lambs were bottle fed.

None of the animals homozygous for either mutation carried the other mutation, implying that no crossover had occurred between the two mutations. The lambs could therefore be divided into six genotype groups, depending on which combination of mutations and wild type allele they carried (Table 2). Homozygous c.960delG-lambs were only produced the second year, since the rams used the first year did not carry this mutation.

The group of homozygous individuals for c.960delG was significantly different from the reference groups, both the wild type (GG_GG) and GG_AA for three of the observed traits (Table 3). The homozygous c.960delG animals had lower daily weaning weight gain (312 g per day), lower weaning weight (44.6 kg), but higher carcass weight (23.3 kg). Daily gain of slaughter weight was very similar for all groups, ranging from 134 to 143 g per day, with no significant differences.

**Table 3:** Solutions ± standard errors for various traits and genotype classes, resulting from mutations at c.960 or c.2360

c.960	GG		
	
c.2360	GG	AG	AA
Weight gain/d from birth to weaning (g)	357 ± 12	352 ± 11	350 ± 12
Weaning weight (kg)	50.1 ± 1.7	49.4 ± 1.6	49.0 ± 1.8
Carcass weight gain/d from birth to slaughter (g)	136 ± 5	134 ± 5	137 ± 5
Carcass weight (kg)	21.4 ± 0.6	21.3 ± 0.6	21.8 ± 0.7
Carcass conformation class (scale 1-15)	7.4 ± 0.3	7.7 ± 0.3	8.1^0.015 ^± 0.4
Carcass fat class (scale 1-15)	6.0 ± 0.3	5.4^0.001 ^± 0.2	5.1^0.000 ^± 0.3

**c.960**	**G(delG)**		**(delG)(delG)**
	
**c.2360**	**GG**	**AG**	**GG**

Weight gain/d from birth to weaning (g)	361 ± 12	349 ± 12	
Weaning weight (kg)	50.2 ± 1.7	48.9 ± 1.8	
Carcass weight gain/d from birth to slaughter (g)	143 ± 5	140 ± 5	142 ± 8
Carcass weight (kg)	22.1 ± 0.6	22.3 ± 0.7	
Carcass conformation class (scale 1-15)	8.3^0.000 ^± 0.3		
Carcass fat class (scale 1-15)	5.0^0.000 ^± 0.3		

Guanine (G) is found at both mutated positions in wild types, while (delG) and adenine (A) respectively, are found when mutations are present. The P-value of genotype classes contrasted with the wild type (GG_GG) is presented as superscript, while the P-value for G(delG)_GG, G(delG)_AG and (delG)(delG)_GG contrasted with GG_AA is given in subscript. The P-values are given only for significant findings (P < 0.05). Solutions are given with the following restrictions; genotype of dam class GG_GG, male, twin and age of dam = 3.

**Figure 1:**
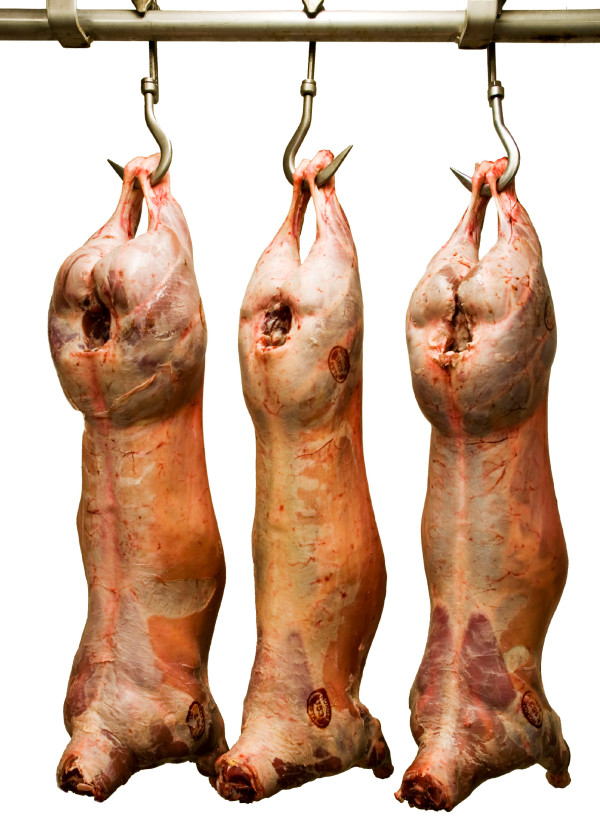
**A typical NWS lamb carcass, flanked by two carcasses homozygous for the MSTN mutation c.960delG**. Carcass weight, EUROP conformation class and fat class (both on a 15 points scale), from the left; 29.5 kg, 15, 4; 18.9 kg, 8, 5, and 24.8 kg, 15, 3. Photo: Audun Flåtten, Animalia.

For carcass conformation and carcass fat, both mutations increased or decreased, respectively, scores in comparison to those of the reference *MSTN *groups numerically (Table 3). For both traits, all genotype groups differed significantly (P < 0.05) from the wild type group (GG_GG), except GG_AG for carcass conformation. For both carcass conformation and carcass fat, the genotype G(delG)_GG was not significantly different from the genotype GG_AA, while the genotypes G(delG)_AG and (delG)(delG)_GG resulted in significant (P < 0.001) effects, towards more meaty and less fatty animals. The wild type group had a carcass conformation class and fat class of 7.4 and 6.0, respectively; homozygotes for the c.2360G>A mutation had 8.1 and 5.1 respectively; and homozygotes for the c.960delG mutation showed the largest effect with 12.5 and 3.0, respectively (for illustration; see Figure 1).

The effect of the ewe's *MSTN*-genotype on her lamb(s) was close to zero and non-significant for all traits (results not shown).

The allelic effects are given in Table 4. The mutation in c.2360 showed a significant additive effect only on carcass conformation (0.3) and fat class (-0.4), and no significant effect of dominance. The mutation in c.960 significantly affected all traits, except for daily carcass weight gain. For this mutation, there were also significant dominance effects for four of these traits. For carcass conformation class, a significant interaction between the mutations was estimated.

**Table 4:** Solutions ± standard errors for various traits and allelic effects

Allelic effect	a2360	d2360	a960	d960	int.
Weight gain/d from birth to weaning (g)	-3 ± 4	-2 ± 5		27^0.001 ^± 8	-7 ± 9
Weaning weight (kg)	-0.6 ± 0.5	-0.2 ± 0.6		2.8^0.004 ^± 1.0	-0.5 ± 1.2
Carcass weight gain/d from birth to slaughter (g)	1 ± 2	-2 ± 3	3 ± 3	4 ± 4	-1 ± 5
Carcass weight (kg)	0.2 ± 0.2	-0.3 ± 0.3		-0.2 ± 0.4	0.3 ± 0.5
Carcass conformation class (scale 1-15)	0.3^0.015 ^± 0.1	-0.1 ± 0.2		-1.7^0.000 ^± 0.3	0.8^0.014 ^± 0.3
Carcass fat class (scale 1-15)	-0.4^0.000 ^± 0.1	-0.2 ± 0.1		0.5^0.010 ^± 0.2	0.0 ± 0.3

Additive (a) and dominance (d) effect for mutations in position c.2360 and c.960 respectively, and the interaction effect (int), when both mutations are present. The P-value of genotype classes contrasted with the wild type (GG_GG) is presented as superscript, while the P-value for G(delG)_GG, G(delG)_AG and (delG)(delG)_GG contrasted with GG_AA is given in subscript. The P-values are given only for significant findings (P < 0.05).

## Discussion

The results show that both the c.2360G>A and c.960delG mutations affect conformation and fat class in NWS lambs, yielding a carcass with less fat and increased muscle mass (Table 3 and 4). The effect of the c.960delG mutation is larger than that of the c.2360G>A mutation. This is in line with the results obtained by Boman et al. [2], who suggest this is most likely due to the different functional impact of the two mutations. The effect of the c.2360G>A mutation, as compared to the wild type, is slightly more pronounced in this experiment, compared to the material reported by Boman et al. [2]. However, in the experiment reported here, we were able to study more than one flock environment, a larger number of lambs in all *MSTN*-groups, and the farmers only partially decided which lambs to slaughter. In addition, the statistical model also accounted for the proper number of lambs following the ewe at weaning, rather than the number of lambs born.

There were no overlap between rams and years. It is possible that the genetic contribution from the rams and the flock-year effects may have been confounded, but this will not affect the relative size of effects of genotype classes. Also, lambs homozygous for the c.960delG mutation were only produced the second year. As the five other genotype classes were produced both years, this lack of complete cross classification should not be a problem.

Since the c.2360G>A-mutation is already segregating in NWS at a medium frequency (Table 2), we hypothesise that in the future this mutation will reach near-fixation in NWS, as in the Texel breed [1,9]. Therefore we tested the other *MSTN *groups against the group homozygous for c.2360G>A, in addition to testing against the wild type.

In Norway, live weight is the most important criterion for deciding when to slaughter lambs. Thus, the higher carcass weight for the homozygous c.960delG mutation group may be explained by enlarged dressing percentage, indicated by the enhanced carcass conformation class for this group (Table 3). The reduced weaning weight and weaning weight gain per day (Table 3) also show that the group homozygous for c.960delG grows slowly. However, it is likely that a possibly enlarged dressing percentage, together with the fact that slaughter information was discarded for slow growing lambs in this group (Table 2), explain why the carcass weight gain per day is closer to that of other groups than expected from live weight gain.

The effects of the c.2360G>A mutation have also been examined in other studies. Before this mutation was reported, Laville et al. [10] had investigated the effect of the corresponding QTL in Belgian Texel sheep. They reported a QTL effect that increased conformation scoring and carcass weight, and reduced the fat score. Kijas et al. [9] had found that under Australian conditions, the g.+6723G>A mutation (equals the c.2360G>A mutation) had significant effects on slaughter measurements of muscling and fatness, but only minor impact on live weight and growth. These results correspond well with our findings.

Similarly, Hadjipavlou et al. [11] had studied the effect of the c.2360G>A mutation on Charollais lambs, and did not find any effect on live weight. With an animal model, AA animals were found to have significantly larger muscle depth than AG and GG animals, while AG and GG animals were not significantly different. None of the fat depths were significantly different. They concluded that the effect on phenotype depended on the genetic background, a point that is clearly demonstrated in our material for carcass conformation class, showing that animals heterozygous for the c.2360G>A mutation are strongly influenced by the genotype at the c.960 position.

## Conclusions

In NWS, increased muscle mass and reduced carcass fat are caused by the c.960delG and the c.2360G>A mutations. The impact of c.960delG is more important compared to c.2360G>A, and displays dominance effects. In the rough grazing environment of this experiment, lambs homozygous for the c.960delG mutation experienced reduced growth rate.

## Competing interests

The authors have been granted a patent in the UK on the diagnostic method of gene testing for the c.960delG mutation (GB2433320).

## Authors' contributions

IAB carried out the experiment, performed the statistical analysis and drafted the manuscript. DIV was responsible for genotyping of the animals, and improved the manuscript, jointly with GK. All authors participated in planning the experiment, read and approved the final manuscript.
